# Cutting Forces during Inconel 718 Orthogonal Turn-Milling

**DOI:** 10.3390/ma14206152

**Published:** 2021-10-16

**Authors:** Agata Felusiak-Czyryca, Marek Madajewski, Paweł Twardowski, Martyna Wiciak-Pikuła

**Affiliations:** Faculty of Mechanical Engineering, Poznan University of Technology, 3 Piotrowo St., 60-965 Poznan, Poland; marek.w.madajewski@doctorate.put.poznan.pl (M.M.); pawel.twardowski@put.poznan.pl (P.T.); martyna.r.wiciak@doctorate.put.poznan.pl (M.W.-P.)

**Keywords:** cutting forces, turn-milling, Inconel 718, tool wear, cutting forces coefficients

## Abstract

Inconel 718 is a material often used in the aerospace and marine industries due to its properties and ability to work in harsh environments. However, its machining is difficult, and therefore methods are sought to facilitate this process. One of such methods is turn-milling. This paper presents the forces during orthogonal turn-milling of the Inconel 718 alloy. In this machining, both the side and the end edge are involved in the material removal, which causes the tool to be more loaded. The forces during turn-milling can be up to 50% higher than in the case of milling, which causes damage to the tool. Tool wear during machining has a significant impact on the values of the cutting force proportional coefficients. In the case of the tested material, it is important to take it into account when creating cutting force models.

## 1. Introduction

Nickel-based superalloys are used in the aviation and marine industries, as parts of gas turbines, parts of space shuttles and nuclear reactors. These are characterized by high strength at elevated temperatures, high abrasive wear resistance and corrosion resistance, as well as significantly higher creep resistance compared to other materials [1]. Inconel alloys belong to the group of the difficult-to-machine materials. This is due to the occurrence of hardening during machining and high temperature in the cutting zone, which ultimately contributes to accelerated tool wear. During machining, an outer-metal chip notch is formed on the edge, which causes deterioration of the machined surface quality [2,3]. During the nickel alloys turning the generated heat continuously affects the edge, drastically accelerating its wear. During milling, the contact of a single edge with the workpiece is variable during rotation, so the temperature influence is not as significant as in turning. There are many research studies on the technology of turn-milling in the literature, but very few of them concern difficult-to-machine materials, so it is impossible to clearly determine the impact of the parameters and machining strategy on the tool life, cutting forces and quality of the produced nickel-based alloys components. In the paper [4], the authors analyzed the effect of various cooling methods and cutting speed on the tool life when machining Inconel 718, Waspaloy and Ti6Al4V. The tool life was higher than in turning for all tested strategies and materials. In the publication [5], the authors analyzed the influence of turn-milling on the roughness and microstructure of the ɣ-TiAl alloy. Boozarpoor et al. [6] in their article analyzed the influence of process parameters such as tool and workpiece rotational speed, feed rate and eccentricity on surface roughness and residual stresses during Inconel 718 turn-milling. The feed had the greatest impact on the tested parameters. They also established the optimal process parameters for the investigated range.

On the other hand, for typical structural materials, such as carbon steels, the authors report that cutting forces during turn-milling are lower than during turning [7,8]. Obtaining such an effect when machining nickel alloys may result in the material surface hardening reduction, and thus increase the durability of the cutting edge. In the article [9] the authors analyzed the influence of the cutting tool inclination on its wear during milling. According to them, the rake angle of 30° the tool wear decreases with the increase of the tool inclination angle. Jin et al. [10] analyzed the tool wear mechanism during orthogonal turn-milling of high strength steel. Mechanism of tool wearing in turn-milling is fatigue-exfoliative wearing, felted wearing in lower cutting speed, and diffuse wearing in higher cutting speed, accompanied with abrasive wearing. The minor flak face wear in turn-milling was higher than in milling and turning.

The literature presents many models describing the forces during orthogonal centric or eccentric turn-milling. In the paper [11], the authors developed an analytical model for orthogonal eccentric turn-milling considering three cases—depending on the position of the tool (different distance from the shaft axis). In two cases, the edges on the tool face are involved in cutting, in the third case only the side edges. The experiments were carried out on AISI 1050 steel. In the paper [12], the researchers created an uncut chip geometry model depending on the machining depth. They notice that for different ranges of the machining depth this cross-section will be different. The model shows significant errors in the component prediction in the *z* direction, while for the *x* and *y* components the mapping is consistent with the experimental data. In the paper [7], the authors used a mechanistic model to predict cutting forces during orthogonal turn-milling. They also defined the relationship between the surface quality and process parameters and suggested an approach of cutting parameters optimization to increase productivity. In the paper [13], the authors investigated the modeling of the cutting forces in orthogonal turn-milling, where a cutter with round inserts has been used. In this paper, the contact area of the tool and the workpiece was determined by the surface mapping method. The most accurate uncut chip geometry model is presented in the article [14]. The authors divided the cutting zone into two types of “small and large depth”. The cutting zone at “large depth” usually occurs during the turn-milling process, while the second type occurs only when the appropriate inequality related to the radius of the tool and workpiece and the depth of cut is met. Their model of forces has an error of 15%. Otalora-Ortega et al. [15] created a numerical model for turn-milling including the eccentricity in the orthogonal turn-milling and the tool geometry for torus and ball milling cutters. The experimental validation results correlate in both tests presenting in the case of the mass errors below 3.5% and the cutting-force errors around 12%. They also found that the wrong tool selection and its eccentricity causes geometric errors on the machined surface.

However, the presented models are not sufficient models for the analyzed process, they do not take into account, incl. tool wear, they are also not models for nickel alloys. When machining nickel alloys, tool wear, depending on its intensity, is a phenomenon that affects the value of the cutting force components F and the dynamics of their changes. Therefore, a model that does not take into account the tool wear would be correct only in the initial stage of the machining and would become unreliable in a relatively short time due to a large error in estimating the force value.

## 2. Materials and Methods

**Figure 1:**
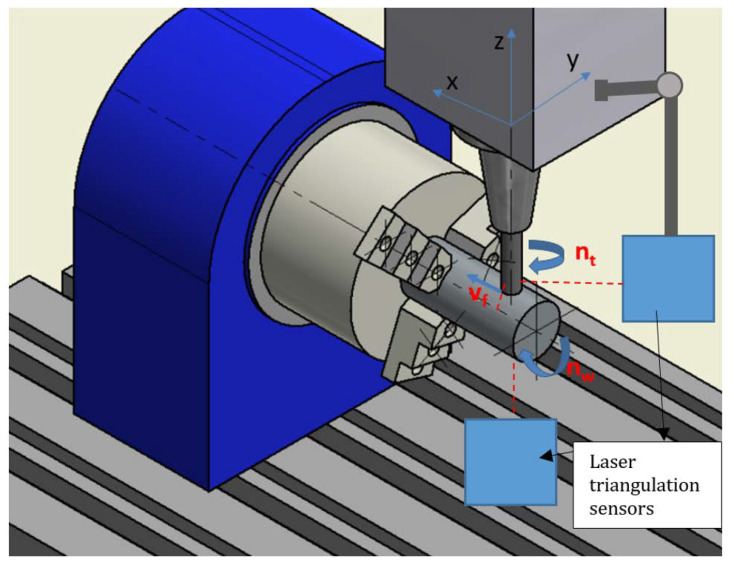
Scheme of the turn-milling station, v_f_—tool movement speed direction, n_t_—tool rotation, n_w_—workpiece rotation.

All tests were carried out on the DMC 70 V hi-dyn three-axis high-speed milling center (Deckel Maho, DMG MORI, Bielefeld, Germany). To enable turn-milling operation, it was equipped with a rotary table (fourth axis) with independent rotational speed control (Figure 1). Thanks to this, it was possible to carry out milling of axially rotating components.

The tests were divided into three phases, in which the stiffness of the configuration was successively determined, a model was created and the cutting force coefficients were derived, in the last part a turn-milling operation was performed to verify the force model. The average forces acting on the cutter in three perpendicular directions were analyzed during the tests. In the tests, SF 440 N 8 × 73.5 × 8 × 12 × 46 × 90 TA carbide cutters from Fanar (Ciechanów, Poland) with a diameter of 8 mm and 4 cutting flutes were used. All the cutters were gripped with the same length—50 mm out of toolholder. The displacement measurement was performed with the use of Micro-Epsilon (Ortenburg, Germany) optoNCDT 1700 laser sensors, linear accuracy 0.08% FSO (Full Span Output), resolution 0.01% FSO, measuring frequency 5 kHz, measuring range 10 mm and laser beam wavelength 670 nm. An Inconel 718 shaft with a diameter of Ø50 mm and a total length of 280 mm was used as the workpiece. Table 1 shows the mass composition of the machined material.

**Figure 2:**
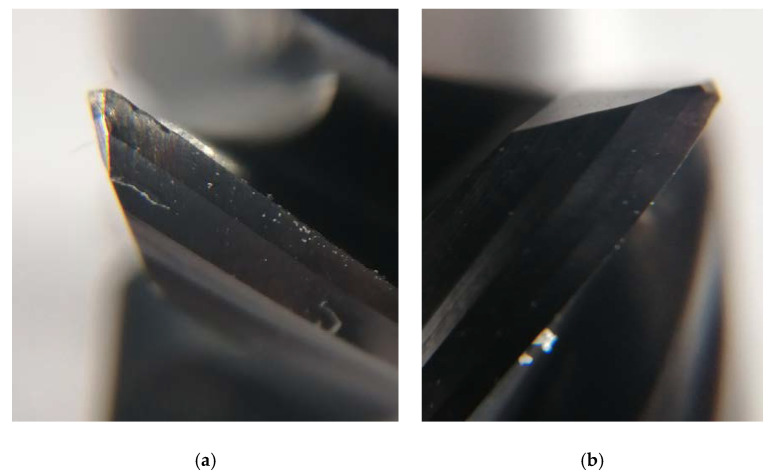
(**a**) Worn and (**b**) new cutting edge. Magnification × 12.

After each pass, the tool wear *VB_B_* (wear width on the flank face) was measured for each edge and the average wear value was calculated. A workshop microscope was used to perform the measurement and photos were taken on the Stereo Discovery V.20 stereo microscope by Zeiss (Jena, Germany) to determine the form of tool wear. Tool wear has two forms here (Figure 2), there is abrasive and adhesive wear resulting from the formation of outer-metal chip notch and its detachment during tool work. 

### 2.1. Kinematic and the Uncut Chip Geometry Model

**Figure 3:**
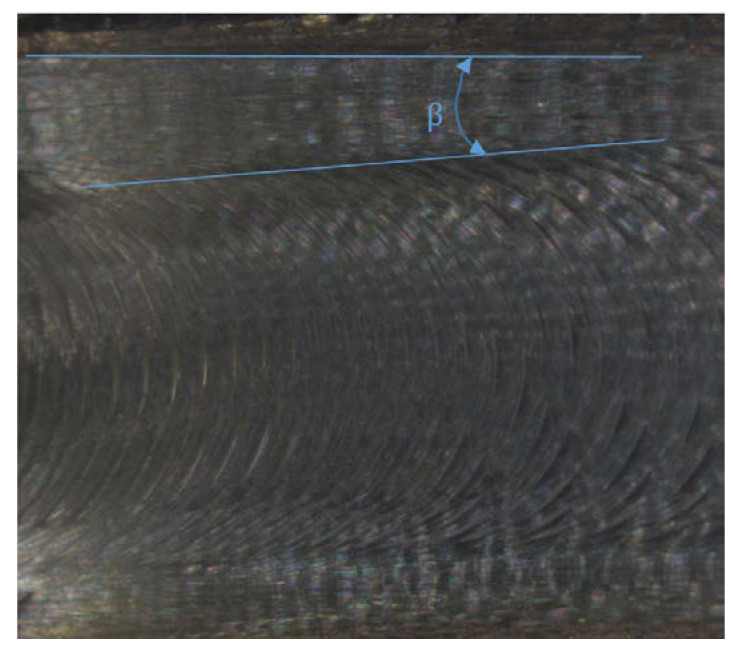
Traces created during turn-milling—magnification × 10.

Unlike milling, turn-milling needs to take into account the workpiece rotation. Thus, the resultant feed is a combination of two movements, the movement of the tool along the axis of the shaft and the movement due to rotation of the workpiece. As a result, the tool moves along the machined surface along a helical line, which can be seen on the machined surface in the form of machining marks. As can be seen in Figure 3, the machining marks are not parallel to the workpiece edge but are arranged diagonally to it at a certain angle β resulting from the combination of these two movements.

To be able to effectively remove the entire allowance, the axial feed per revolution of the workpiece must not exceed the tool radius. The following formulas (1)–(5) describe the turn-milling kinematic:

(1) $$v_{c}=\frac{2\pi r_{t}n_{t}}{1000}$$

*v_a_ = f_a_n_w_* (2)

*v_t_ = 2πR_w_n_w_* (3)

*f_t_ = 2πR_w_* (4)

(5) $$f_{z}=\frac{n_{w}\sqrt{f_{a}^{2}+f_{t}^{2}}}{n_{t}z}$$

where:

*v_c_*—cutting speed [m/min]
*v_a_*—axial feed speeed [mm/min]
*v_t_*—tangential feed speed [mm/min]
*f_t_*—tangential feed [mm]
*f_a_*—axial feed [mm/workpiece rotation]
*f_z_*—feed per tooth [mm]
*n_t_*—tool rotational speed [rpm]
*n_w_*—workpiece rotational speed [rpm]
$r_{t}$—tool radius [mm]
*R_w_*—workpiece radius [mm]

The uncut chip geometry model was calculated based on the formulas from article [14].

The measurements were carried out at a constant cutting speed *v_c_* = 50 m/min and a constant cutting depth *a_p_* = 1.5 mm and variable feed rate per tooth (Table 2). 

### 2.2. Stiffness Determination

**Figure 4:**
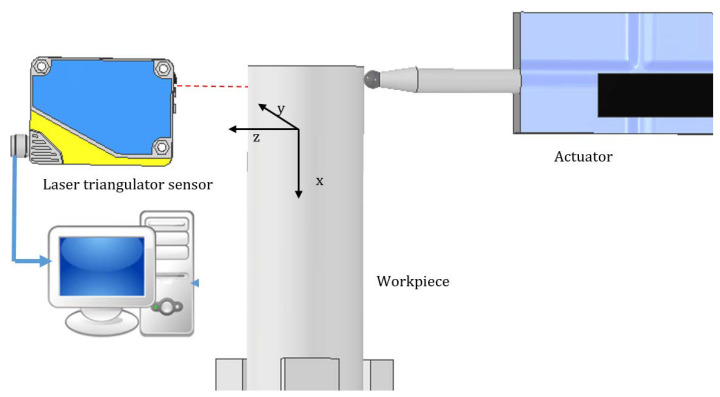
Scheme of the workpiece measuring station along the *z* axis.

**Figure 5:**
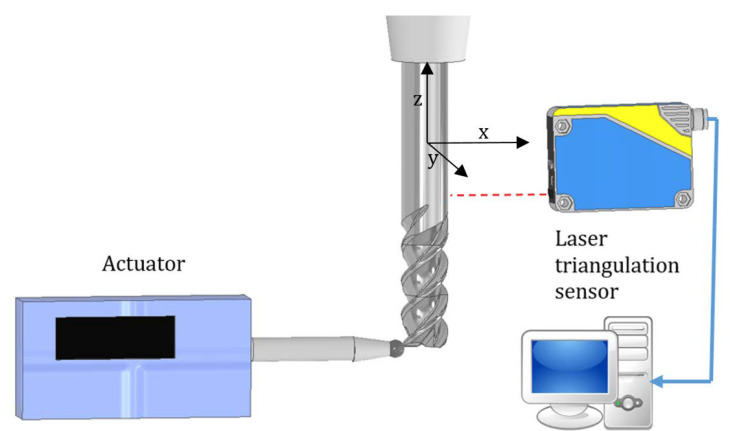
Scheme of the milling cutter measuring station in the *x* and *y* axes.

The stiffness in the *z* direction complies with the stiffness of the workpiece system (Figure 4), and in the *x* and *y* direction with the tool system, measured as in Figure 5 in the radial direction. In the further research, the displacement obtained from the measurements was multiplied by the stiffness, thus obtaining the values of the average cutting forces acting on the system.

In the first phase, the stiffness of the tool and workpiece system was determined. For this purpose, a known force ranging from 0 to 1000 N was applied to the tool and the workpiece. During the loading, the displacement was measured and then, using formula (6), the stiffness of the system was calculated.

(6) $$\bar{x}=\frac{\Sigma \frac{F_{1}}{a_{1}}+\frac{F_{2}}{a_{2}}+\ldots \frac{F_{n}}{a_{n}}}{n}\;;n\in N$$

where:

$\bar{x}$—average stifness [N/µm]
*F*—applied force [N]
*a*—linear displacement [µm]
*n*—number of cases

On this basis, the following average values of stiffness were obtained:

*x_z_* = 2.367 [N/µm]
*x_x,y_* = 3.474 [N/µm].

### 2.3. Calculating Cutting Forces Coefficions

**Figure 6:**
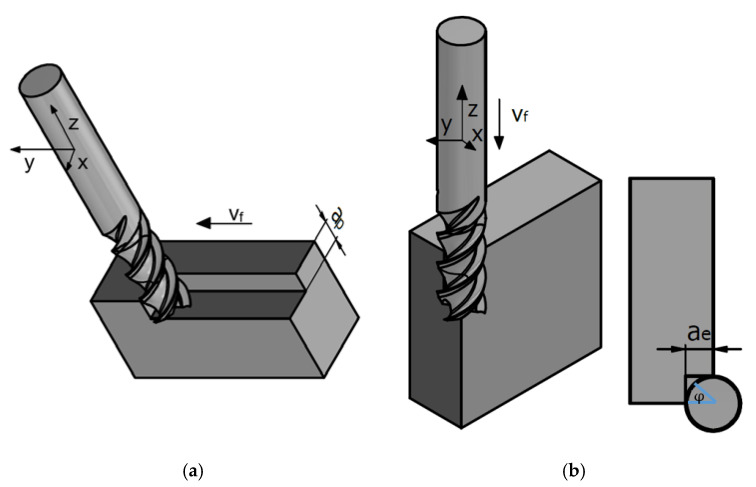
Kinematics of (**a**) slot and (**b**) plunge milling.

According to the forces model (7)–(9), the coefficients of proportionality of the cutting forces should be determined. These coefficients are calibrated mechanically, separately for the flank and end edge of the milling cutter. This is necessary due to the different geometry of those two edges. To calculate the cutting force coefficients *K_c_* and *K_e_*, tests were carried out in two feed directions (radial and axial milling—Figure 6), because in the considered process, both cutting edges are involved in milling.

*dF_rq_ = K_rc_ e (φ,r) + K_re_)dr* (7)

*dF_tq_ = K_tc_ e (φ,r) + K_te_)dr* (8)

*dF_aq_ = K_ac_ e (φ,r) + K_ae_)dr* (9)

*dF_rq_*—differential of radial force [N]
*dF_tq_*—differential of tangential force [N]
*dF_aq_*—differential of axial force [N]
*K_rc_, K_re_, Ktc, K_te_, K_ac_, K_ae_*—cutting force coefficients [N/mm^2^]
*e (φ, r)*—instantaneous chip thickness [mm]

(10) $$K_{tcF}=\frac{4\bar{F_{yc}}}{za_{p}}$$

(11) $$\;K_{rcS}=\frac{-4\bar{F_{xc}}}{za_{p}}$$

(12) $$K_{acF}=\frac{\pi \bar{F_{zc}}}{za_{p}}$$

(13) $$K_{teF}=\frac{\pi \bar{F_{yc}}}{za_{p}}$$

(14) $$K_{reF}=\frac{-\pi \bar{F_{xc}}}{za_{p}}$$

(15) $$K_{aeF}=\frac{2\bar{F_{zc}}}{za_{p}}$$

(16) $$K_{tcE}=\frac{-\bar{F_{xc}}\left(\pi^{2}+2\right)+\pi \bar{F_{yc}}}{\frac{za_{e}}{8\pi }\left(\pi^{2}+4\right)}$$

(17) $$K_{rcE}=\frac{\pi \bar{F_{xc}}-2\bar{F_{yc}}}{\frac{za_{e}}{8\pi }\left(\pi^{2}+4\right)}$$

(18) $$K_{acE}=\frac{2\pi \bar{F_{zc}}}{za_{e}}$$

(19) $$K_{teE}=\frac{-2\pi \bar{F_{xe}}}{za_{e}}$$

(20) $$K_{reE}=\frac{2\pi \bar{F_{ye}}}{za_{e}}$$

(21) $$K_{aeE}=\frac{4\bar{F_{ze}}}{za_{e}}$$

### 2.4. Cutting Forces

**Figure 7:**
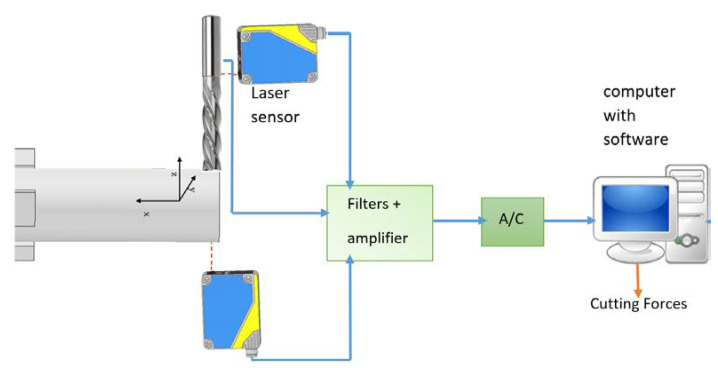
Scheme of determining cutting forces during turn-milling.

The cutting forces during the milling and turn-milling process were determined from the measured deflection and the known stiffness as in Figure 7, with the accuracy control of the determined values by simultaneous measurement with a piezoelectric dynamometer during milling.

## 3. Results

### 3.1. Uncut Chip Geometry Model

**Figure 8:**
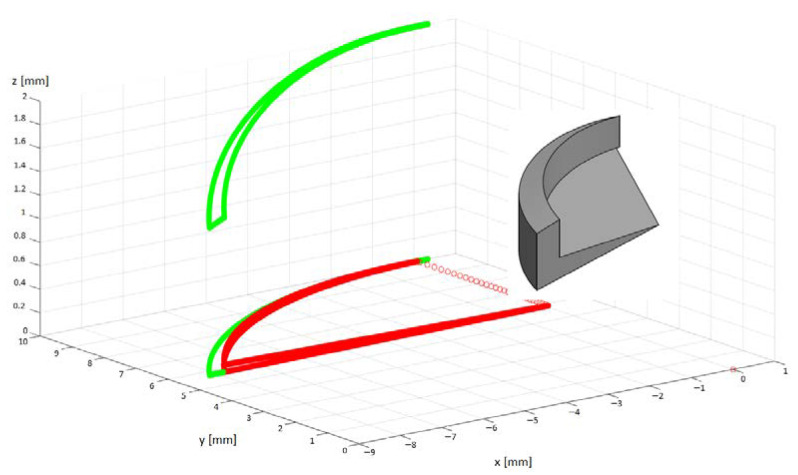
3D representation of the uncut chip geometry, green-flank uncut chip area and red end uncut chip area.

Figure 3 shows a graphical representation of the analytical approach. This representation of the uncut chip geometry has been programmed to draw the flank zone in green, and the lines in red belong to the end milling zone. The approach presented in these studies applies only to cutters with negligible corner radius. To be able to model the geometry in the MatLab program (Natick, MA, USA), the discretization of the cutter rotation angle *φ* every 0.01° was used. When creating the uncut chip geometry models, the cutting parameters were used as in Table 2. In the visible model (Figure 8), for the shaft rotational speed *n_w_* = 10 [rpm], the tool rotational speed *n_t_* = 1990 [rpm], the feed per tooth *f_z_* = 0.2 mm and the cutting depth *ap* = 1.5 mm, the angle of entry for the side zone (green) was 150° (in relation to the *y* axis), the exit angle for the side zone was 82.15°, the exit angle for the end zone was 84°.

### 3.2. Cutting Forces Coefficions

For each variant of milling, a series of tests was carried out with variable feed values per tooth, constant cutting speed and constant depth or width of cut. The end edge is assumed to be straight along the radius of the cutter. The values of milling coefficients were calculated according to the formulas (10)–(15) for slot-milling and (16)–(21) for plunge milling for different wear values *VB_B_* (wear width on the flank face). These formulas were developed based on the general formula from the book [16]. *K_qe_* coefficients related to elastic and plastic strains and frictional phenomena in the contact area of the flank surface and the workpiece. *K_qc_* factors are related to shear.

The average cutting forces were expressed by the linear function described by the formula (22) and graphs 9 and 10.

*F_q_ = F_qc_ f_z_ + F_qe_* (22)

where *q* is *x*, *y*, *z*
where *Fqc* is the slope of the linear function and *F_qe_* is the constant value.

Expressing the forces using a linear function allows experimental determination of the cutting force coefficients.

**Figure 9:**
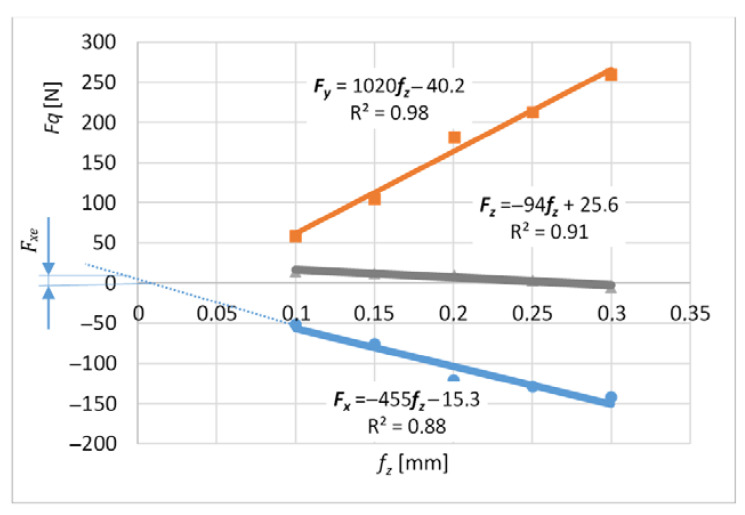
Graph of the average cutting forces for slot-milling.

**Figure 10:**
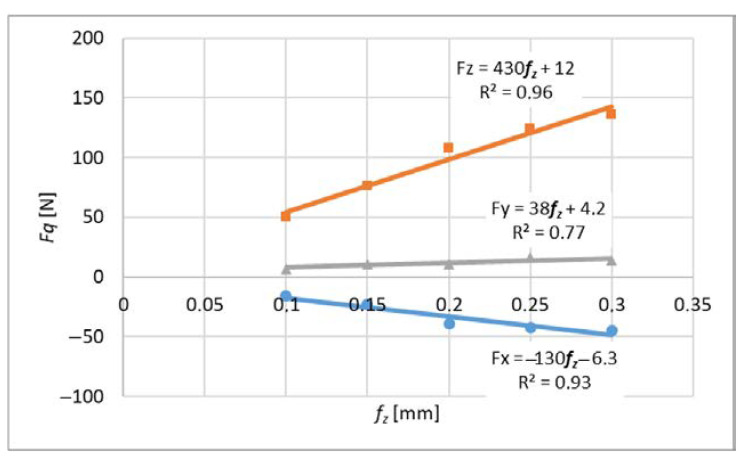
Graph of the average cutting forces for plunge milling.

**Figure 11:**
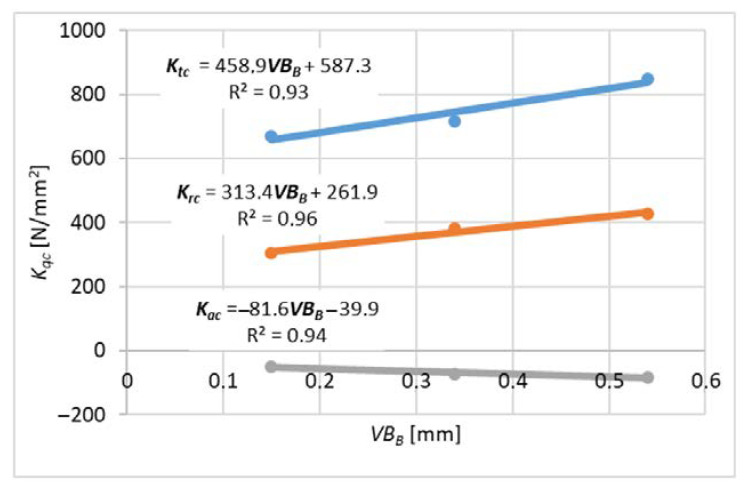
Effect of tool wear on the cutting force coefficient *K_c_* for slot-milling.

**Figure 12:**
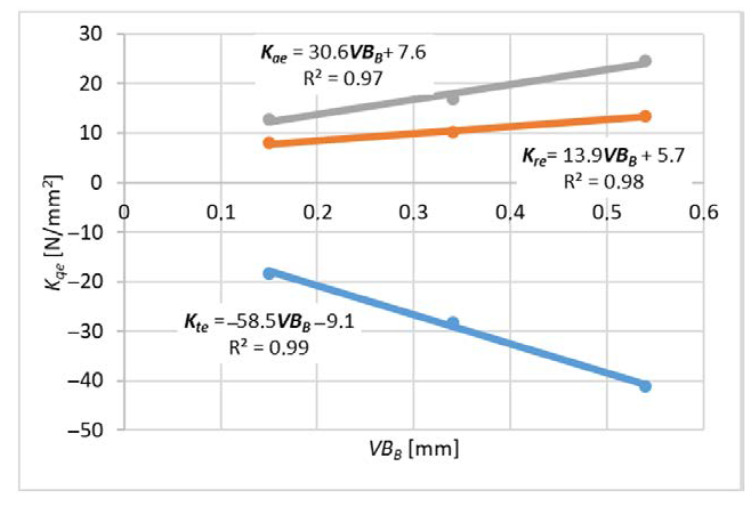
The influence of wear on the coefficient of cutting forces *K_e_* for slot-milling.

**Figure 13:**
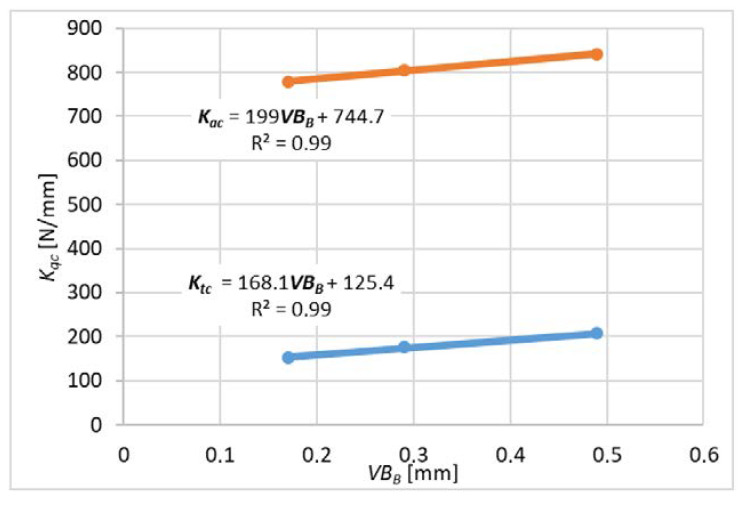
The influence of tool wear on the cutting force coefficient *K_c_* for plunge milling.

**Figure 14:**
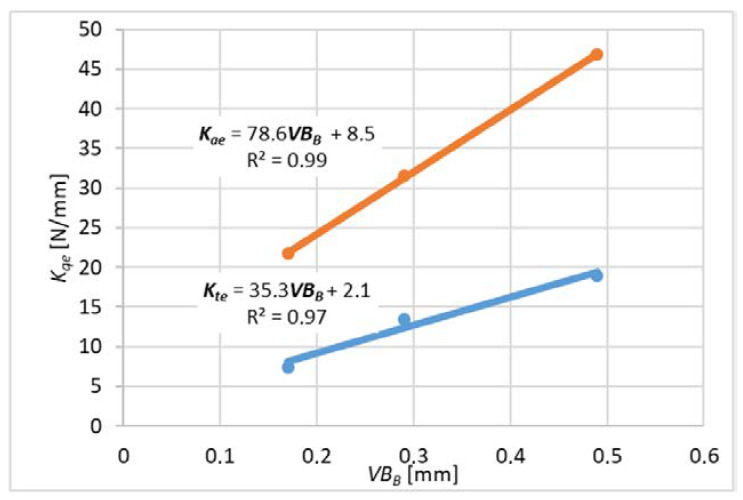
The Influence of tool wear on the cutting forces coefficient *K_e_* for plunge milling.

Measurements for each feed value were repeated 3 times, which made it possible to analyze the impact of tool wear on the cutting force coefficients. A higher number of repetitions was not possible due to the short life of the tools. For each value of the feed per tooth in the first trial, new cutters were used (based on these results, Figure 9 and Figure 10 were obtained), then the measurements were repeated with the same cutters to determine the effect of tool wear on the values of cutting coefficients (Figure 11, Figure 12, Figure 13 and Figure 14). The figures show example diagrams of the dependence of forces on the feed for slot (Figure 9) and plunge milling (Figure 10). The figure also shows the value of the friction force *F_xe_*.

Then, an analysis of the variability of the cutting force proportionality coefficients was carried out as in Figure 11, Figure 12, Figure 13 and Figure 14 due to tool wear. As can be seen, the values of the coefficients increase with the increase of tool wear. Inconel milling forces are strongly correlated with wear. The chip notch affects not only tool wear but also directly the forces because it temporarily changes the shape of the cutting edge. As wear progresses, bigger outer-metal chip notch edge occurs at the edge, which can lead to exceeding tool strength and brittle fracture.

It can be concluded that the increase in tool flank wear *VB_B_* caused a linear increase in the absolute values of the cutting force coefficients. This observation indicates that the progressive wear (flank wear *VB_B_*) increases the contact length of the tool and the workpiece, which in turn causes an increase in the force value.

### 3.3. Research and Analysis for Turn-Milling

**Figure 15:**
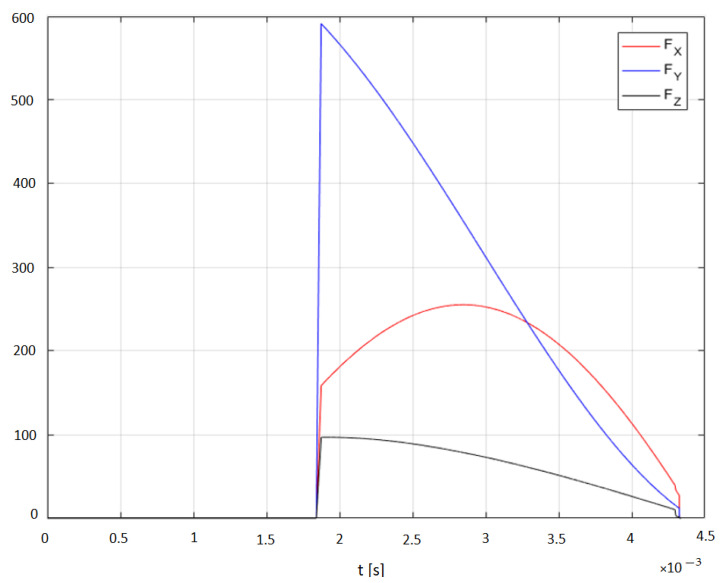
Predicted cutting forces.

**Figure 16:**
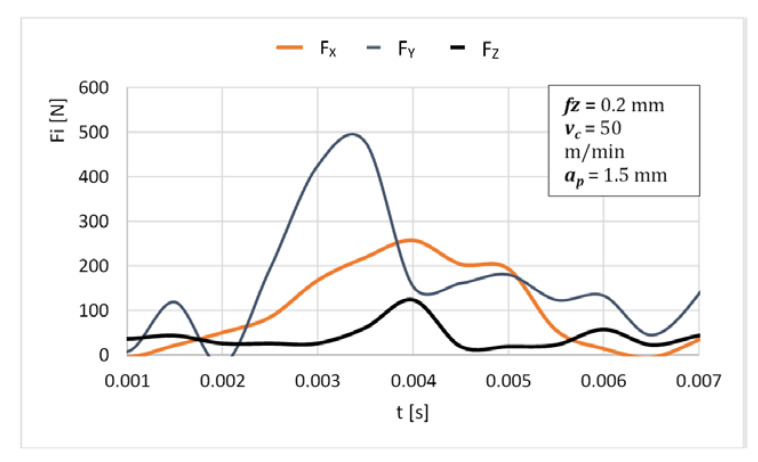
Actual cutting forces.

Based on the determined uncut chip geometry and the calculated coefficients, the values of the cutting forces were determined (Figure 15), and then compared with the actual values measured during orthogonal turn-milling (Figure 16). The results shown represent the forces for one edge. The same tools and cutting parameters were used in the verification tests as in the previous tests.

**Figure 17:**
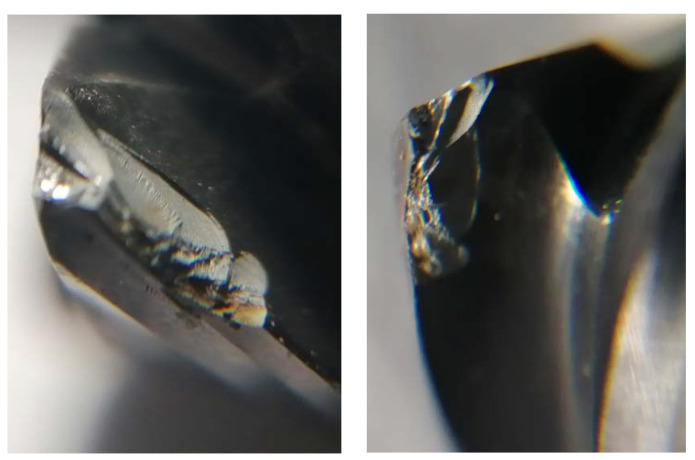
Brittle fracture tool wear occurring during machining. Magnification × 12.

Comparing the actual and the determined waveforms, it can be seen that these distributions are similar to each other, and the differences in the maximum values are on average about 11% for the *Fx* force, about 17% for the *Fy* force and about 16% for the *Fz* force. The forces are not measured directly, but determined based on displacement, which affects the inaccuracy. It should be noted that the forces occurring in the *x* and *y* directions during turn-milling are significantly greater than the forces occurred in the same directions during milling. The greater forces acting on the edges, the proportion of the end cutting edge and the uncut chip geometry on the cutting edges appearing during machining Inconel leads to brittle fracture tool wear in the form of breakage of the cutting edges or the entire tool (Figure 17).

**Figure 18:**
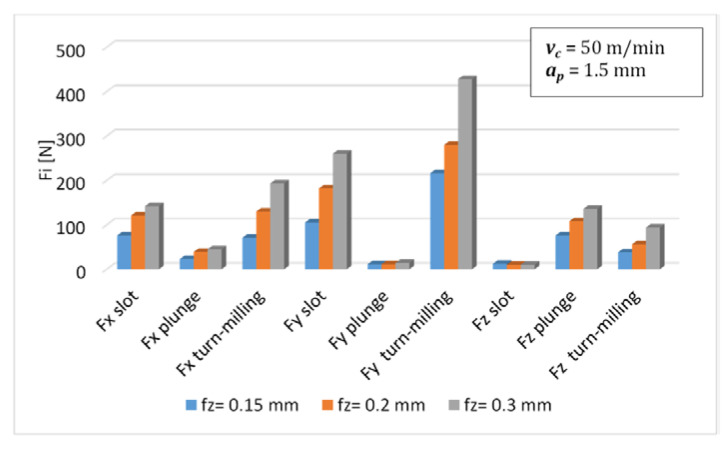
The influence of feed *fz* on forces for different machining types.

The influence of the tooth feed on the cutting forces for each of the previous machining types was analyzed. The results were compared on the graph (Figure 18). In the case of the *Fx* force, its values are similar between slot-milling and turn-milling; however, at the highest feed, the *Fx* turn-milling force increases significantly. A significant increase can also be observed in the other directions. With these parameters, brittle fracture of the edge also occurred most often, and it should be assumed that it is not possible to perform machining with these parameters. Regardless of the machining strategy, the impact of changing the feed value on the force *Fz* was low. The increase in force *Fy* during turn-milling at the highest feed rate is greatest and increases by approximately 50%.

## 4. Conclusions

Based on the above research, the following conclusions can be drawn:

- The forces occurring during the turn-milling process are higher than in the case of conventional milling (with the same cutting parameters), which may lead to the tool damage during machining. This fact should be taken into account when selecting the process parameters. During the tur-milling process, it is not recommended to use a feed per tooth 0.3 mm. The smallest forces were obtained with a feed 0.15 mm.
- For the milling cutter used, the cutting force coefficients of the flank and end edge are different and therefore must be calibrated individually.
- The simulated results shows that under certain machining conditions, both the flank and leading edge contribute significantly to the total cutting forces.
- Tool wear during machining has a significant impact on the values of the cutting force proportional coefficients. In the case of the tested material, it is important to take it into account when creating cutting force models.

## Figures and Tables

**Table 1:** Mass fractions of elements in Inconel 718 alloy.

	Ni	Cr	Nb	Mo	Ti	Al	Co	Si
Min.%	50	17	4.75	2.8	0.65	0.2	-	-
Max.%	55	21	5.50	3.30	1.15	0.80	1.00	0.35
	**Mn**	**Cu**	**C**	**P**	**S**	**B**	**Fe**	
Min.%	-	-	-	-	-	-	-	
Max.%	0.35	0.30	0.08	0.015	0.015	0.006	balance	

**Table 2:** Cutting parameters.

lp.	*v_c_* [m/min]	*a_p_* [mm]/*a_e_* [mm]	*f_z_* [mm]
1.	50	1.5	0.1
2.	50	1.5	0.15
3.	50	1.5	0.2
4.	50	1.5	0.25
5.	50	1.5	0.3

## Data Availability

The data presented in this study are available on request from the corresponding author.
